# Demographics, clinical features, and comorbidities of high-altitude polycythaemia: a multicentre, retrospective, observational study

**DOI:** 10.7189/jogh.16.04042

**Published:** 2026-01-30

**Authors:** Mei Yang, Yuxuan Zhu, Lin Liu, Chuanliang Pan, Lifen Li, Qingqing Su, Wenwen Zhou, Linxi Fu, Lin Yang, Fengming Luo, Lei Chen

**Affiliations:** 1 Department of High Altitude Medicine, Center for High Altitude Medicine, West China Hospital of Sichuan University, Chengdu, Sichuan, China; 2 High Altitude Medicine Key Laboratory of Sichuan Province, West China Hospital of Sichuan University, Chengdu, Sichuan, China; 3 Department of Pulmonary and Critical Care Medicine, West China Hospital of Sichuan University, Chengdu, Sichuan, China; 4 Department of Pharmacy, Personalized Drug Therapy Key Laboratory of Sichuan Province, Chengdu, Sichuan Academy of Medical Science & Sichuan Provincial People’s Hospital, Sichuan, China; 5 Department of Pharmacy, People’s Hospital of Aba Tibetan and Qiang Autonomous Prefecture, Aba, Sichuan, China; 6 Department of Pulmonary and Critical Care Medicine, 363 Hospital, Chengdu, Sichuan, China; 7 Department of Critical Care Medicine, People’s Hospital of Litang County, Litang, Sichuan, China; 8 Department of Gastroenterology, People’s Hospital of Aba Tibetan and Qiang Autonomous Prefecture, Aba, Sichuan, China

## Abstract

**Background:**

High altitude polycythaemia (HAPC) has posed a major burden due to its high prevalence and multisystem involvement among highlanders, but clinical data on HAPC is scarce. We aimed to describe the clinical characteristics of patients with HAPC in China.

**Methods:**

Adult patients diagnosed with HAPC in five hospitals of China between August 2012 to May 2024 were retrospectively enrolled. We analysed information including demographics, living altitude, haemoglobin concentration (Hb) and comorbidities, and fitted restricted cubic splines models with multivariable adjustments to investigate the relationship between age, altitude and Hb.

**Results:**

A total of 1098 HAPC patients were included and 97 individuals of them did not provide information on ethnicity. Of the remaining 1001 participants, 93% were native Tibetans. The median Hb showed a significant difference (*P* < 0.0001) between male (21.9 g/dL, interquartile range (IQR) = 21.4–22.9 g/dL) and female patients (19.6 g/dL, IQR = 19.2–20.8 g/dL), and was slightly higher in Tibetans than Han migrants, especially in females (19.6 g/dL, IQR = 19.2–20.7 g/dL *vs*. 19.3 g/dL, IQR = 19.2–19.5 g/dL) (*P* = 0.198). Restricted cubic splines models revealed Hb exhibited a positive linear correlation with altitude (*P*-overall = 0.027, *P*-nonlinear = 0.291), with a rate of 0.3g/dL/1000 m of elevation, whereas no significant relationship with age (*P*-overall = 0.974, *P*-nonlinear = 0.860). The commonest comorbidities were hypertension (18.5%) and pneumonia (17.6%). Besides, heart failure (*P* < 0.001), chronic airway disease (*P* = 0.018) and pulmonary heart disease (*P* < 0.001) were more prominent in females while liver disease (*P* = 0.079) was more frequent in males.

**Conclusions:**

This study suggests a much higher proportion of HAPC in native Tibetans, and the Hb in HAPC patients remains significant gender-specific and altitude-dependent variations. Moreover, in addition to hypertension and pneumonia, gender-specific comorbidity surveillance should pay attention to digestive system disease in male HAPC patients and cardiopulmonary system disease in female HAPC patients.

High altitude is generally considered as an area exceeding 2500 m above the sea level [1]. The unique high-altitude environment characterised by reduced atmospheric pressure and oxygen leads to a range of hypoxia-related disorders [2]. Individuals who spend extended periods at high altitude are prone to have chronic mountain sickness, the hallmark feature of which is excessive erythrocytosis (EE), also widely referred to high altitude polycythaemia (HAPC) [1,3]. From a pathophysiological perspective, HAPC is the result of physiological adjustments to decreased oxygen availability, involving a series of gene regulation, epigenetic modification, inflammatory and endocrine responses, which has not been fully understood [4–7].

Over 140 million people reside at high altitudes worldwide, including over 80 million in Asia and approximately 35 million in South America [1,8]. It is estimated that the prevalence of HAPC is 4.5%–34% in the Andes, 5.6% in Han Chinese migrants and 1.2% in native Tibetans [8–10]. Robust evidence has demonstrated positive relationships of HAPC with increased blood viscosity and risk of thrombosis, which further cause a series of disorders affecting multiple organ systems, particularly cardiopulmonary system, such as pulmonary hypertension and adverse cardiovascular events [11–14]. It is worth noting that, given the growing number of lowlanders migrating to highlands due to work and tourism worldwide, HAPC has been a burgeoning threat to highland dwellers’ health and posed marked burden on the health care system [15,16]. The treatments for HAPC and HAPC-related diseases are currently limited by the interpretations of disease characteristics and mechanisms. Understanding the clinical features of HAPC could be helpful in optimising prevention and control strategies.

Evidence has suggested that characteristics of highlanders differed by gender and ethnicity, such as the haemoglobin concentration (Hb) and variations [8,17,18]. Previous studies mainly targeted on the general high-altitude residents, whereas data on those with HAPC, especially Tibetans, an Asian population with significant heterogeneity compared to other highlanders, were scarce [19,20]. Therefore, to provide data of Asian HAPC, we conducted a multicentre, retrospective, observational study in the Sichuan province. Sichuan province is located in the southwest of China, straddling the Qinghai-Tibet Plateau, with basins, plains, hills, and plateaus. High-altitude residents in Sichuan mainly comprise native Tibetans, Han migrants and Yi ethnic population. We aimed to describe the demographics, clinical features and comorbidities of patients with HAPC.

## METHODS

### Study design and patients

In this study, we retrospectively screened and included patients diagnosed with HAPC between August 2012 to May 2024, from five hospitals in Sichuan province: West China Hospital of Sichuan University, Sichuan Provincial People’s Hospital, People’s Hospital of Aba Tibetan and Qiang Autonomous Prefecture, People’s Hospital of Litang County and 363 Hospital. Participants were enrolled according to the following criteria:

1) age ≥18 years;

2) full-time or long-term (≥10 years) resident at an elevation above 2500 m;

3) diagnosed with HAPC by qualified physicians in the five hospitals, based on the Consensus Statement on Chronic and Subacute High Altitude Diseases (females Hb ≥19 g/dL and males Hb ≥21 g/dL) [1].

Pregnant individuals or those receiving any treatment affecting Hb, such as recombinant human erythropoietin and iron supplementation, were excluded.

This study was conducted in accordance with the Strengthening the Reporting of Observational Studies in Epidemiology reporting guidelines for observational studies and the Declaration of Helsinki. The study protocol was approved by the Ethics Committee of West China Hospital of Sichuan University (No. 2023–1164).

### Data collection

We reviewed medical records of eligible participants. The information of patients was picked up using a standardised collection form, including age, gender, ethnicity, residential address, medical history, comorbidity and baseline blood Hb. All the comorbidities were diagnosed and coded by qualified physicians in the five study centres, according to the Simplified Chinese version of International Classification of Diseases-10 (ICD10-CN) criteria, which is based on the original version of ICD10 published by World Health Organization and has been extensively adopted by hospitals and clinics throughout China [21–23].

### Statistical analysis

Descriptive data were expressed as number of patients (percentage) for categorical variable, mean with standard deviation (SD) or median with interquartile range (IQR) for continuous variables. For test of variable normality, we combined the histogram, Q-Q diagram and the Shapiro-Wilk test. Blood Hb was compared across patient groups stratified by gender, age (18–29, 30–39, 40–49, 50–59, 60–69, 70–79 and ≥80 years) and altitude (2500–3000, 3000–3500, 3500–4000 and ≥4000 m), using the independent two-sample t, Mann-Whitney U, one-way analysis of variance or Kruskal-Wallis H-tests, as appropriate. We employed multivariable restricted cubic spline (RCS) regression to examine the relationships of age and altitude with Hb, as these continuous exposures may exhibit nonlinear associations that cannot be adequately captured by simple linear terms. This approach allows for flexible modelling of potential non-monotonic relationships while avoiding strong parametric assumptions. We performed graphical diagnostics by plotting the Residual *vs*. Fitted Values and partial residual plots for age and altitude, and assessed model fit through comparing the RCS model to a linear model using Likelihood Ratio Test (LRT). According to the methodological recommendations in the field of biostatistics and epidemiology, we compared the Akaike information criterion (AIC) and Bayesian information criterion (BIC) of RCS models with varying knot numbers (three, four, and five) to determine the optimal functional form for continuous predictors. The model demonstrating optimal fit based on AIC and BIC was selected for final presentation. Besides, we also conducted sensitivity analyses by running RCS models using three knots (5th, 50th, and 95th percentiles), four knots (at the 5th, 35th, 65th, and 95th percentiles) and five knots (at the 5th, 27.5th, 50th, 72.5th, and 95th percentiles) to test the robustness of results. All models were adjusted for gender, age, altitude, hypertension and pneumonia. *P* < 0.05 was considered significant. Statistical analyses were performed using *R* software, version 4.4.0 (R Core Team, Auckland, New Zealand, 2020) and GraphPad Prism software version 8.0 (GraphPad Software Inc., San Diego, CA, USA).

### Patient and public involvement

As this study applied a retrospective and observational design, patients or the public were not involved in the design, or conduct, or reporting of our research.

## RESULTS

### Participants characteristics

A total of 1098 patients were included, with 876 males and 222 females. The mean ages of male and female patients were 48 years (SD = 13) and 54 years (SD = 17) respectively. There were 97 individuals not providing information on ethnicity. Of the remaining 1001 participants, a large proportion were native Tibetans (93%) while the Han migrants accounted for only 6%. The average living altitude for all individuals was 3596.7 m (SD = 457.9), and 87% of them were residing at an elevation above 3000 m. For male patients, the average altitude was 3607 m (SD = 455.6) and for females was 3556 m (SD = 465.3) (**Table 1**).

**Table 1:** Characteristics of study population*

Characteristics	Overall (n = 1098)	Males (n = 876)	Females (n = 222)	*P-*value†
Age (years)	49 ± 14	48 ± 13	54 ± 17	<0.001
Ethnicity (%)‡				
*Tibetan*	927 (92.6)	727 (91.9)	200 (95.2)	0.445
*Han*	60 (6.0)	52 (6.6)	8 (3.8)	
*Other ethnic groups*	14 (1.4)	12 (1.5)	2 (1.0)	
Altitude (metres)	3596.7 ± 457.9	3607.0 ± 455.6	3556.0 ± 465.3	0.140
Haemoglobin level (g/dL)	21.7 (21.1–22.6)	21.9 (21.4–22.9)	19.6 (19.2–20.8)	<0.001
Haemoglobin level – stratified by age (years)				
*18–29*	21.6 (21.0–22.5)	22.0 (21.5–22.8)	20.0 (19.3–21.0)	<0.001
*30–39*	21.7 (21.2–22.6)	21.9 (21.4–22.8)	19.4 (19.1–20.0)	
*40–49*	21.9 (21.2–22.8)	22.0 (21.4–22.9)	19.5 (19.2–20.1)	
*50–59*	21.8 (21.1–22.8)	21.9 (21.4–23.2)	19.9 (19.3–20.9)	
*60–69*	21.6 (21.1–22.4)	21.9 (21.4–22.9)	19.5 (19.2–20.5)	
*70–79*	21.3 (20.2–22.4)	21.7 (21.2–22.8)	19.6 (19.3–20.4)	
*≥80*	21.3 (21.0–21.6)	21.4 (21.3–21.8)	21.1 (19.5–21.5)	
Haemoglobin level – stratified by altitude (meters)				
*2500–3000*	21.6 (21.1–22.5)	21.6 (21.3–22.6)	19.6 (19.3–21.2)	<0.001
*3000–4000*	21.6 (21.1–22.5)	21.9 (21.4–22.8)	19.6 (19.3–20.6)	
*≥4000*	21.8 (21.2–22.9)	22.0 (21.4–23.0)	19.8 (19.2–21.1)	
Comorbidities (%)	1059 (96.4)	839 (95.8)	220 (99.1)	0.017
*Hypertension*	203 (18.5)	159 (18.2)	44 (19.8)	0.567
*Pneumonia*§	193 (17.6)	151 (17.2)	42 (18.9)	0.557
*Heart failure*	105 (9.6)	63 (7.2)	42 (18.9)	<0.001
*Liver disease*	103 (9.4)	89 (10.2)	14 (6.3)	0.079
*Pulmonary heart disease*	63 (5.7)	34 (3.9)	29 (13.1)	<0.001
*Cerebrovascular disease*	62 (5.6)	48 (5.5)	14 (6.3)	0.634
*Chronic airway disease¶*	98 (8.9)	63 (7.2)	35 (15.8)	0.018
*Thrombosis*	40 (3.6)	31 (3.5)	9 (4.1)	0.714

HAPC – high-altitude polycythaemia, COPD – chronic obstructive pulmonary disease.

*Data are presented as mean ± SD, median (interquartile range) or n (%).

†Males *vs*. females.

‡97 participants without information on ethnicity were not included.

§Pneumonia related to mycobacteria was not included.

¶Including chronic obstructive pulmonary disease, chronic bronchitis, bronchiectasis and asthma.

### Variations in haemoglobin concentration

**Figure 1:**
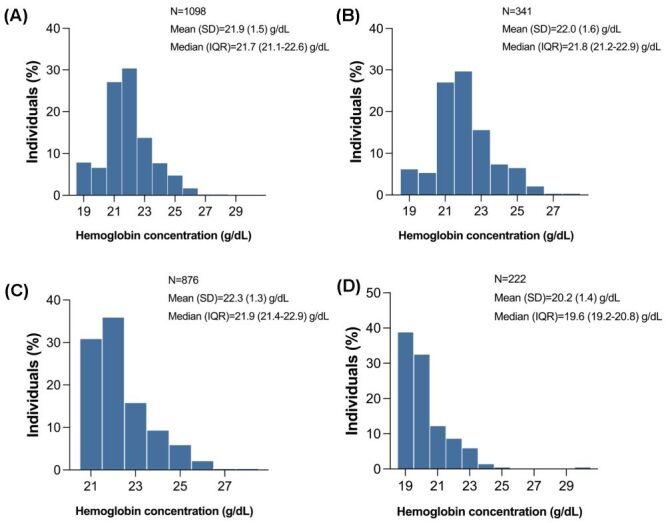
Frequency distribution of haemoglobin concentration in patients with high-altitude polycythaemia. **Panel A.** All participants. **Panel B.** Participants living at an altitude above 4000 m. **Panel C.** Male patients. **Panel D.** Female patients. IQR – interquartile range, SD – standard deviation.

Among all participants, Hb ranged from 19.4 to 29.5 g/dL and displayed a right-skewed distribution, with a median concentration of 21.7 g/dL (IQR = 21.1–22.6 g/dL), similar to those living at an elevation above 4000 m (21.8 g/dL, IQR = 21.2–22.9 g/dL) (**Figure 1**, Panels A–B). Significant difference (*P* < 0.0001) (**Table 1**) was observed between male (median = 21.9 g/dL, IQR = 21.4–22.9 g/dL) and female patients (median = 19.6 g/dL, IQR = 19.2–20.8 g/dL) (**Figure 1**, Panels C–D). Besides, the median Hb was slightly higher in Tibetans than Han migrants, no matter in males (21.9 g/dL, IQR = 21.4–22.9 g/dL *vs*. 21.8 g/dL, IQR = 21.2–22.6 g/dL) (*P* = 0.299) and females (19.6 g/dL, IQR = 19.2–20.7 g/dL *vs*. 19.3 g/dL, IQR = 19.2–19.5 g/dL) (*P* = 0.198).

**Figure 2:**
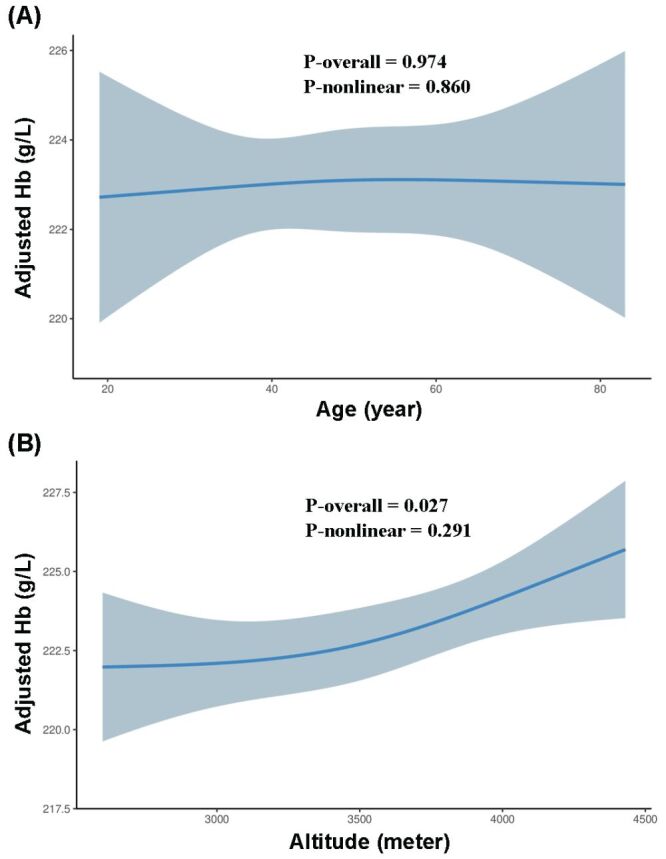
Association of haemoglobin concentration with age (**Panel A**) and altitude (**Panel B**). The restricted cubic splines models are adjusted for gender, age, altitude, hypertension and pneumonia. The grey band represents the 95% confidence interval for the smooth curve.

The RCS models were applied to assess association between age, altitude and Hb. For graphical diagnostics of models, partial residual plot for age exhibited approximately linear pattern, with no evidence of substantial curvature or systematic deviations, while the partial residual plot for altitude exhibited positive association between altitude and Hb (Figure S1–2 in the **Online Supplementary Document**). Similarly, residuals *vs*. fitted values plots demonstrated random scatter around zero with constant variance across the range of predicted values, indicating no violations of key model assumptions (Figure S3–4 in the **Online Supplementary Document**). Besides, comparison of the RCS model with linear model using LRT revealed no statistically significant results for either age (χ^2^ = 0.031, df = 1.000, *P* = 0.860) or altitude (χ^2^ = 1.122, df = 1.000, *P* = 0.289). Despite this, the RCS models provided valuable insights into the nature of age-Hb and altitude-Hb relationships. Compared to four-knot and five-knot models, the three-knot RCS model revealed the lowest AIC and BIC and was selected for the final presentation (Table S1 in the **Online Supplementary Document**). According to three-knot RCS models, after adjusting for gender, altitude, hypertension and pneumonia, no significant relation was shown between age and Hb (*P*-overall = 0.974, *P*-nonlinear = 0.860) (**Figure 2**, Panel A), whereas a positive linear relationship was observed between altitude and Hb (*P*-overall = 0.027, *P*-nonlinear = 0.291) (**Figure 2**, Panel B), with an effect value of 1.517 g/L for 514 m (Table S2 in the **Online Supplementary Document**), indicating the rate of increase in Hb with altitude was 0.3g/dL/1000 m. No gender difference was observed in the age-Hb (*P* = 0.130) and altitude-Hb (*P* = 0.527) relationships. Additionally, in the multivariable RCS models, female gender (*P* < 0.001) and pneumonia (*P* = 0.016) was negatively associated with, whereas hypertension (*P* = 0.676) was not correlated with Hb. Sensitivity analyses for four-knot and five-knot models revealed consistent results (Table S2 in the **Online Supplementary Document**).

### Comorbidities of HAPC

**Figure 3:**
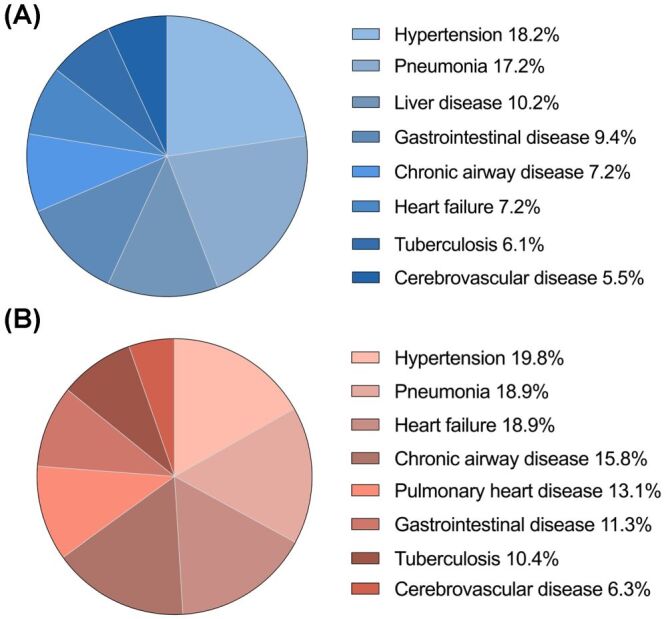
Comorbidities of patients with high-altitude polycythaemia. **Panel A.** Male patients. **Panel B.** Female patients. Chronic airway disease: chronic obstructive pulmonary disease, chronic bronchitis, bronchiectasis or asthma. Pneumonia: mycobacterial infection was not included.

The incidence of comorbidities differed between both genders and was generally higher in female patients (females 99.1% *vs*. males 95.8%, *P* = 0.017). Hypertension was the commonest comorbidity, occurring in 18.2% of males and 19.8% of females (*P* = 0.567), lower than previously reported in highland dwellers [24–27], followed by non-mycobacterial pneumonia, with a prevalence of 17.2% in males and 18.9% in females respectively (*P* = 0.557). Besides, cardiopulmonary, liver and gastrointestinal diseases were also frequent (**Figure 3**, Panels A–B). By comparison, heart failure (females 18.9% *vs*. males 7.2%, *P* < 0.001), chronic airway disease (females 15.8% *vs*. males 7.2%, *P* = 0.018) and pulmonary heart disease (females 13.1% *vs*. males 3.9%, *P* < 0.001) were more prevalent in female patients, while liver disease was more common in male patients (males 10.2% *vs*. females 6.3%, *P* = 0.079) (**Table 1**, Table S3–5 in the **Online Supplementary Document**).

## DISCUSSION

This study highlighted clinical characteristics of patients with HAPC, with a focus on Hb variations and comorbidities. The study population primarily consisted of adult Tibetans in plateaus of Sichuan, with an average residing altitude of 3597 m. The main findings included a right-skewed, gender- and potential ethnicity-stratified distribution of Hb, which was notably higher in males, slightly higher in Tibetans compared to Han migrants, and also correlated linearly with altitude. Moreover, the comorbidity profile revealed apparent gender-specific disparity, and a lower-than-expected incidence of hypertension, alongside an inverse association of pneumonia with Hb.

Although the LRT indicated that the RCS models did not provide statistically superior fit compared to linear specifications, our decision to employ this flexible modelling approach was grounded in several important methodological considerations. First, the use of RCS represents a conservative analytical strategy that avoids imposing potentially incorrect linearity assumptions a priori. By allowing for potential nonlinearity while ultimately demonstrating linear relationship, we provided stronger evidence for the true nature of these associations than would be available from simply assuming linearity from the outset. This approach minimises the risk of both type I errors (from forcing nonlinear relationships into linear frameworks) and type II errors (from overfitting truly linear trends). Second, the non-significant LRT, combined with the linear patterns observed in the partial residual plots, provide coherent and mutually reinforcing evidence that the relationships are appropriately characterised as linear. This convergence of graphical and statistical evidence enhance confidence in our conclusions regarding the essentially linear nature of these associations. Finally, our approach exemplifies contemporary methodological standards in epidemiological research, where flexible modelling techniques are increasingly recommended for the analysis of continuous exposures. By transparently reporting both the RCS results and the formal comparisons with linear models, we provide complete information to evaluate the robustness of our findings.

A relatively stable high-altitude population has long resided in Sichuan province because of its specific geographical location, comprising native Tibetans, Yi population and Han migrants etc. In the five centres of our study, two are located in Tibetan regions, with average elevations of 2500 m (Aba Prefecture) and 4300 m (Ganzi Prefecture), and the other three centres also have considerable proportions of Tibetan patients. This could be a main explanation for the predominance of Tibetan patients in our study.

Evidence has suggested that Hb varies markedly across different ethnicities [8]. Himalayan highlanders were reported having a mean Hb of 1.4 g/dL lower than Andean highlanders [17]. Max Gassmann and colleagues applied a multiple-regression model to estimate Hb for different populations and found that compared with the USA reference group, the increase of Hb with altitude (g/dL/1000 m) in South American residents was nearly four times higher than African people and almost 22 times higher than their Asian counterparts [18]. For Asian population, Hb in native Tibetans was lower than other highlanders, such as Chinese Han migrants and Indian Tamils [28]. Based on the 2005 consensus of International Working Group, the prevalence of EE among Andean population ranged from 4.5 to 34% [9,10], and was about 5.6% across Han migrants while as low as 1.2–2.6% in Tibetans [29,30]. According to the World Health Organization 2008 criteria [31], the prevalence of EE in Tibetans was also markedly lower than migrants [32,33]. As the oldest indigenous mountainous population, native Tibetans have exhibited better haematological adaptation to hypoxic environment, considered as a result of natural selection of genes [19,28]. Genomic studies highlighted that EPAS1/HIF2A and EGLN1/PHD2, both involved in the HIF pathway, had undergone adaptive mutations in Tibetans [34,35]. EPAS1/HIF2A was reported to lower Hb by regulating the erythropoietin production [35–37]. EGLN1/PHD2 mediated physiological responses to hypoxia (including erythropoiesis) through the degradation of HIFs [35,37]. Other genetic variations have also been identified, but need further validation [34,35,38].

It remains unclear whether patients with HAPC exhibit similar ethnic patterns in Hb. Based on current data, we found that ethnic disparity possibly persisted in HAPC patients. The median Hb of Tibetan participants was slightly higher than Han migrants, especially in female patients (Tibetans 19.6 g/dL *vs*. Han migrants 19.3 g/dL). Moreover, two small-sample studies [39,40] even reported higher mean Hb of 23.0 g/dL and 22.8 g/dL in male Tibetan patients than our finding (22.3 g/dL, n = 727). These results were in contrast to that in the male patients (22.0 g/dL) from Andes (South America) at similar altitude [10]. However, the robustness of these ethnic disparities requires further verification through both demographic and genetic investigations.

Previously reported gender difference in Hb across highlanders remained among those with HAPC. The main explanations could be sex hormone and metabolic disparities, as testosterone was indicated favouring highland adaptation and EE [41–43]. Besides, gender discrepancy regarding the age-Hb association has been revealed in the general population and healthy individuals [44–46]. After the age of 40, Hb tended to decrease in males whereas increase in females [44,45]. Similar trends were shown in our study population (**Table 1**). However, gender difference was not significantly observed in the multivariable-adjusted age-Hb and altitude-Hb relationships, suggesting the difference may also be influenced by other confounding factors, such as altitude, age and comorbidities, *etc*. Meanwhile, the positive association between altitude and Hb has been recognised, but the magnitude of increase varies across ethnicities [18,47,48]. Evidence highlighted that Hb exhibited an altitude-dependent increase at a rate of 1 g/dL/1000 m for Andean population whereas in Tibetans it was 0.68 g/dL/1000 m) [18]. In our study, the rate of 0.3g/dL/1000 m indicated that this magnitude further decreased in Tibetans with HAPC, which may be correlated with the combined effects of higher baseline Hb and body’s compensatory mechanisms, leaving less room for further elevation. However, due to limited sample size, we were unable to fit models separately for male and female patients, thus large-scale studies are warranted to validate these findings.

Ambulatory blood pressure has been indicated to increase progressively with elevating altitude after short-term highland exposure [49]. The main physiological mechanism is that hypoxia stimulates the peripheral chemoreceptors, thereby triggering sympathetic activation, which leads to higher heart rate, elevated cardiac output and blood pressure [50]. For the long-term dwellers in high altitude, such as Tibetans, studies have shown a weak positive correlation between blood pressure and altitude [51,52], possibly induced by increased blood viscosity and polycythaemia [50,51]. Clinical evidence also suggested that polycythaemia remained a strong determinant of hypertension after multivariate adjustments except altitude [27,53–55]. The prevalence of hypertension was 44.7% in the Chinese general population [56], 15.2–51.2% in Tibetans [24,26,57] and 27.1–54.5% in other highlanders [25,27,52], with marked heterogeneity. However, in our study, although hypertension was the commonest comorbidity of HAPC, the prevalence of which was apparently lower than previously reported in the general population and highlanders, and no statistical relationship was identified between Hb and hypertension after multivariable adjustments including altitude. These findings may further reflect the long-term complex adaptation mechanisms of blood pressure to high altitude in highlanders (especially Tibetans) and besides, altitude as the determinant of atmospheric pressure and oxygen concentration, exerts confounding effect on the correlation between Hb and hypertension. Previous evidence supports our findings to some extent. Ruiz L and colleagues highlighted that the prevalence of hypertension was lower in Peruvian highlanders than sea level dwellers and a prolonged altitude stay could ameliorate systemic hypertension [58]. Suppression of the renin-angiotensin-aldo-sterone system and loss of plasma volume at high altitude may also contribute to the lower blood pressure of Tibetans with HAPC in our study [49,50]. Future studies should elucidate the long-term and complex dynamics of blood pressure and their underlying mechanisms under high-altitude conditions, and whether Tibetans, other high-altitude and low-altitude populations exhibit divergent adaptation patterns in blood pressure, or manifest persistent differential responses to hypobaric hypoxia.

Another interesting finding of our study was the comorbidity of pneumonia. Ranking as the second commonest comorbidity, pneumonia was found negatively related to Hb. This unexpected association reflects a complex interaction where acute infections modulate erythropoiesis. Lifestyle factors and high-altitude environment exposure may predispose Tibetan residents to infections [59]. However, because the lack of robust epidemiological data on incidence of pneumonia in the general Tibetan population, we could not perform conclusive comparison across subgroups. It is worth noting that, compared to healthy highland controls, HAPC patients were reported having elevated inflammatory cytokine levels including IL-1β, IL-2, IL-3, and TNF-α [60], suggesting the presence of clinical or subclinical infections. Whether this phenomenon having genetic regulatory underpinnings remains unclear. Additionally, although male patients have higher Hb, their cardiopulmonary responses appear less pronounced, with lower incidence of cardiopulmonary disorders compared to females. This may be explained by inherent sex differences in lung structure and function under normal and specific disease states [61]. The higher frequency of liver disease among male patients may be explained by Tibetan dietary pattern, in which alcohol intake is an important component [62].

Our study provides Hb data for HAPC patients (especially Tibetans) across different genders, ages and altitudes, thereby laying a foundation for future investigations on high-altitude populations. Besides, the comorbidity patterns may carry implications for future research, clinical practice and health care planning in high altitudes. First, initial attention should be paid on hypertension and pneumonia in HAPC patients. Monitoring and managing blood pressure and respiratory infections might be integral to HAPC care. Second, the identified gender-specific disparities suggested a differentiated approach to clinical surveillance. The routine check-ups for HAPC patients could be optimised: females may benefit from more rigorous cardiac and pulmonary function assessments, while males should receive enhanced screening for liver function. Finally, from a public health perspective, the substantial burden of cardiopulmonary comorbidities, coupled with the unique needs of the Tibetan population, highlighted the necessity for allocating specialised health care resources to high altitude clinics. This includes training for managing complex HAPC cases and ensuring the availability of diagnostic tools for early detection of these predominant comorbidities.

However, several limitations should be noted. First, the restriction of our patient recruitment to hospitals within Sichuan Province could limit the generalisability of our findings to a wider HAPC population and constitutes potential selection bias. The majority of study participants are Tibetans, thus data on Han migrants and other indigenous highlanders such as Yi population, are still lacking. Second, given the retrospective and multi-centre study design, inherent risk of data incompleteness is inevitable. For example, because of limited data, we failed not analyse relationships between Hb and other important factors, such as smoking, stage of pregnancy and ethnicity, as well as their confounding effects [63]. And there were also 97 participants not providing information on ethnicity. These could compromise the accuracy of the results. Notably, we specifically improved this issue through rigorous data collection and inclusion criteria. The primary variables of interest, Hb and comorbidities, were also essential for patient diagnosis and management at the participating hospitals. All comorbidities were systematically diagnosed and coded by qualified physicians at each centre according to the standardised ICD-10-CN criteria. Therefore, for these key variables, data were complete across all included cases. Nonetheless, we cannot entirely rule out the possibility of unmeasured confounding factors or variations in clinical documentation practices across different centres. Third, as the Hb data represent a single baseline measurement taken at presentation, they are subject to potential influence from acute factors and may not accurately represent the stable, chronic state, thus prospective and longitudinal data on Hb are required for validation.

## CONCLUSIONS

This study identifies a much higher proportion of HAPC in native Tibetans from Sichuan, China, and findings on Hb of HAPC show significant gender and altitude-specific variations. Comorbidity profiles revealed hypertension and pneumonia as the most frequent concurrent conditions, as well as significant gender differences: females showed a higher burden of cardiopulmonary disease, whereas liver disease was more prevalent in males. These results underscore the potential need for clinicians to implement gender-differentiated and altitude-aware screening and management strategies for HAPC, and for public health practitioners to integrate these metrics into surveillance systems. Future research is needed to prioritise large-sample, multi-centre studies to validate these findings, complemented by longitudinal follow-up and genetic investigations to elucidate the underlying mechanisms.

## Additional material


Online Supplementary Document
