# Supplementary material for: Demographics, clinical features, and comorbidities of high-altitude polycythaemia: a multicentre, retrospective, observational study
Source: J Glob Health. 2026 Jan 30;16:04042. doi: 10.7189/jogh.16.04042 (PMC12856962; doi:10.7189/jogh.16.04042)
Supplement: Online Supplementary Document [file jogh-16-04042-s001.pdf]

Supplement to: Yang M, Zhu Y, Liu L, Pan C, Li L, Su Q, Zhou W, Fu L, Yang L, Luo F, Chen L. Demographics, clinical features, and comorbidities of high-altitude polycythaemia: a multicentre, retrospective, observational study. J Glob Health. 2026;16:04042.

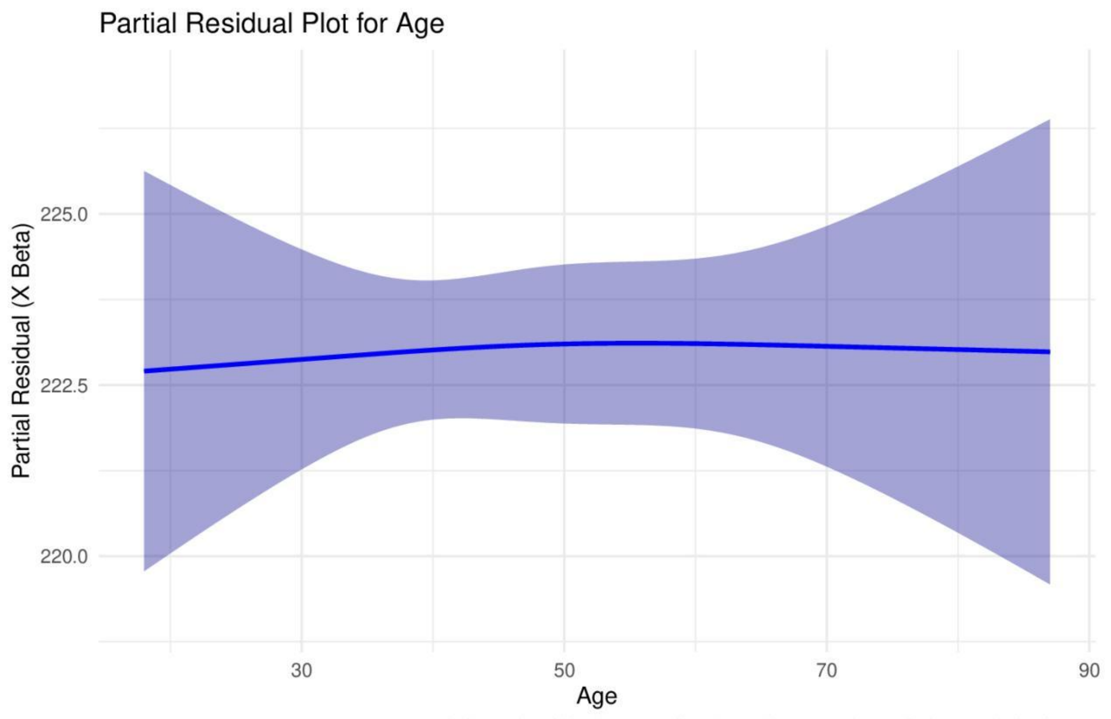

Figure S1. Partial residual plot for age in the restricted cubic spline model, adjusted for altitude, gender, hypertension and pneumonia. The gray band represents the 95% confidence interval for the smooth curve.

Visual inspection of the partial residual plot for age revealed no discernible non-linear pattern, with the loess smooth curve nearly indistinguishable from a straight line. This graphical finding was consistent with our formal statistical test, which showed no significant non-linearity (P = 0.860) and non-significant association overall (P = 0.974).

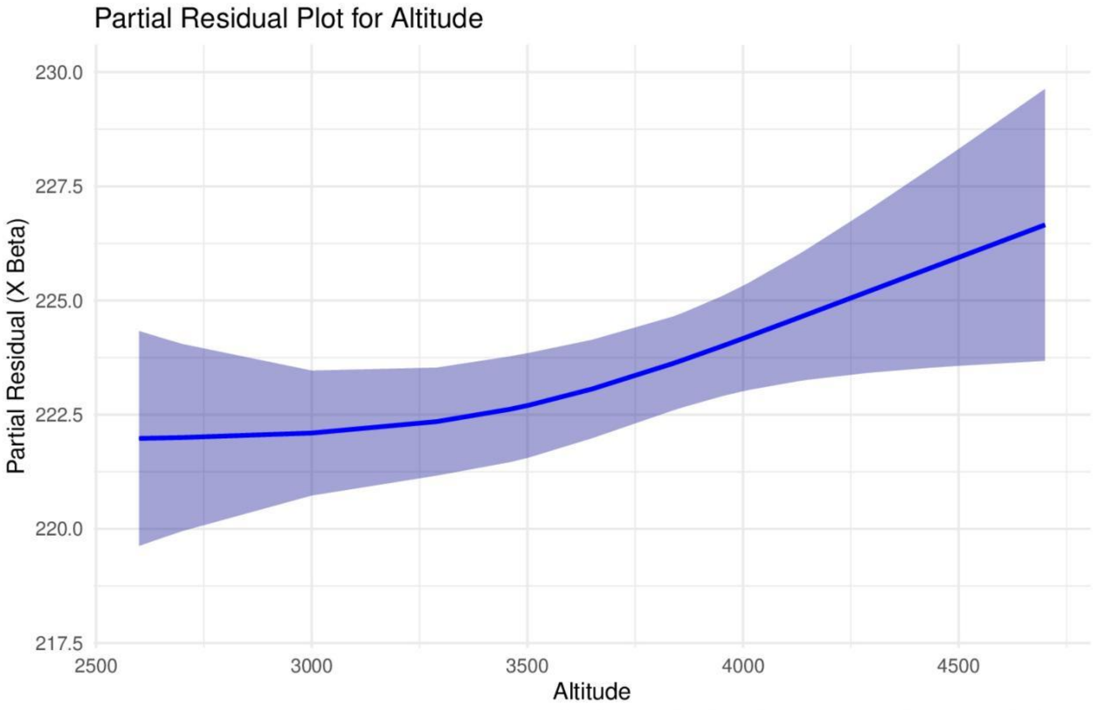

Figure S2. Partial residual plot for altitude in the restricted cubic spline model, adjusted for age, gender, hypertension and pneumonia. The gray band represents the 95% confidence interval for the smooth curve.

The smoothly increasing curve illustrates the positive association between altitude and hemoglobin concentration. The non-significant test for non-linearity (P = 0.291) supports the essentially linear nature of this relationship, while the significant overall test (P = 0.027) confirms its statistical reliability.

(A)

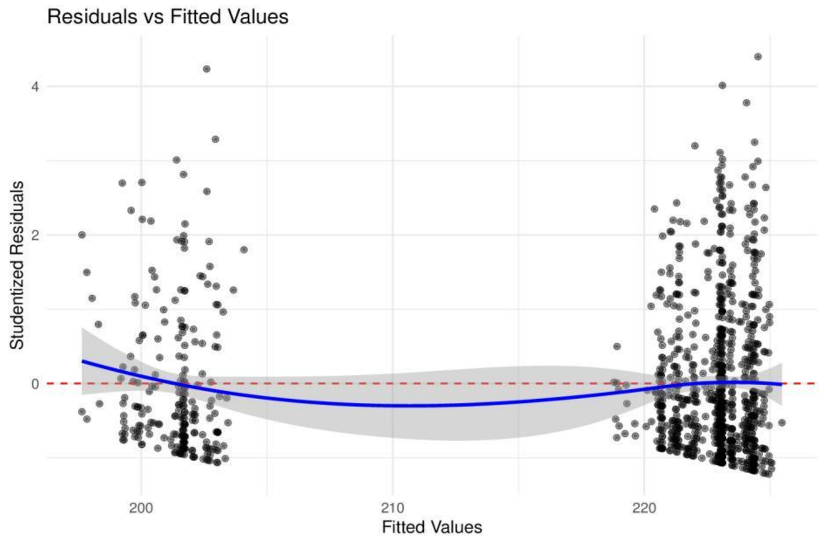

(B)

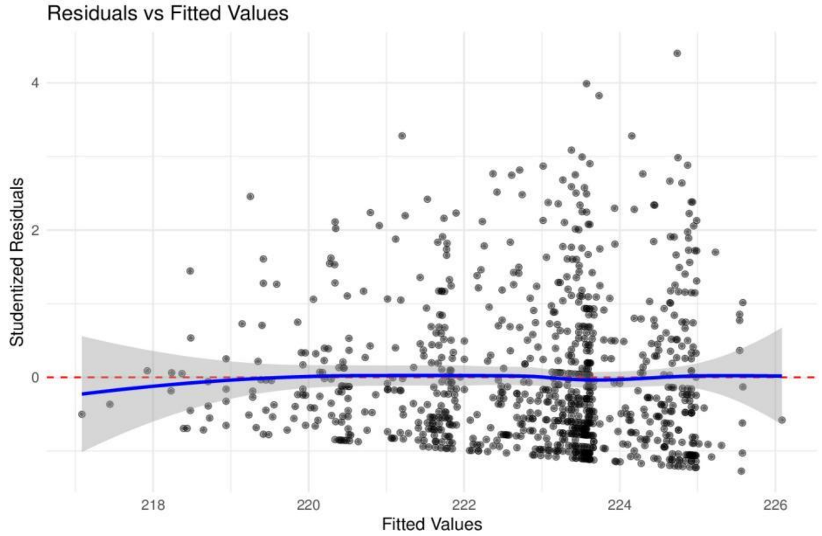

(C)

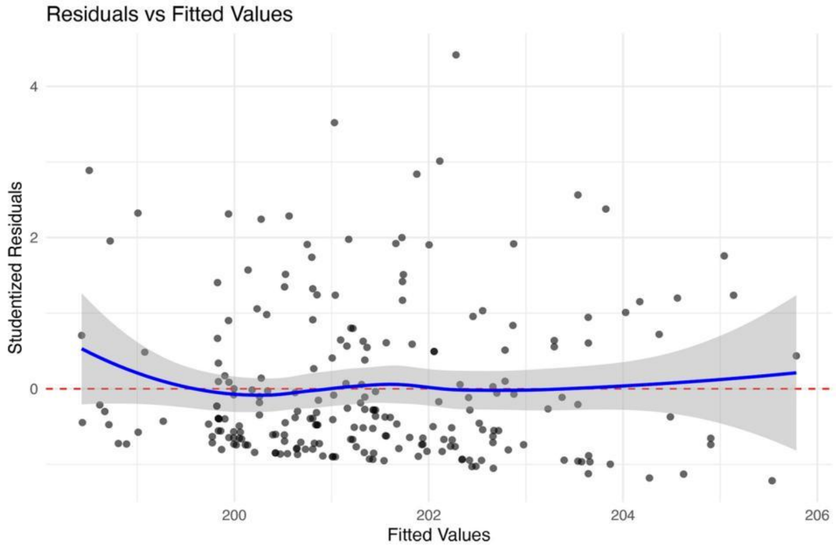

Figure S3. Residuals versus Fitted Values for age in the restricted cubic spline model,

adjusted for altitude, gender, hypertension and pneumonia. **Panel A.** All participants. **Panel B.** Male patients. **Panel C.** Female patients. The gray band represents the 95% confidence interval for the smooth curve.

In the **Panel A**, residual diagnostics for the model revealed a pattern characterized by two vertical clusters of points on either side of zero. The loess smooth curve exhibited a slight dip in the central region, which was a geometric artifact of smoothing across these two distinct subgroups. This finding did not indicate model misspecification but rather confirmed that the model adequately captured the major source of heterogeneity in the data. This pattern was attributable to the strong, dichotomous effect of gender on hemoglobin concentration because when we verified it separately in males (**Panel B**) and females (**Panel C**), this pattern no longer existed.

In the **Panel B**, the residual plot in males exhibited higher point density at higher fitted values, mirroring the greater representation of older individuals in our male samples.

The parallel-to-zero pattern of the smooth curves in males and females demonstrated that our models provided unbiased predictions throughout the age range, despite the uneven sampling across different age groups.

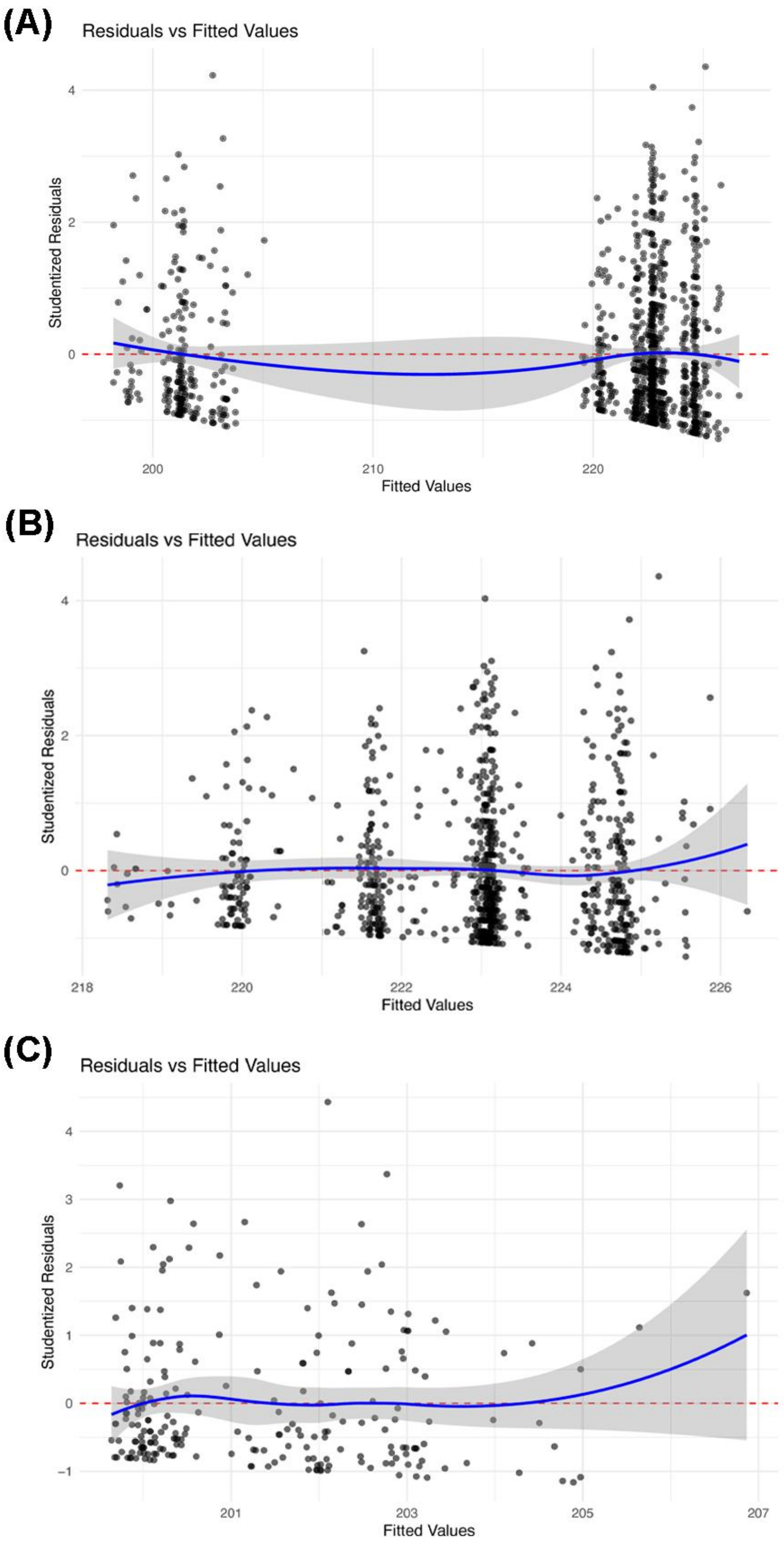

Figure S4. Residuals versus Fitted Values for altitude in the restricted cubic spline

model, adjusted for age, gender, hypertension and pneumonia. **Panel A.** All participants. **Panel B.** Male patients. **Panel C.** Female patients. The gray band represents the 95% confidence interval for the smooth curve.

In the **Panel A**, residual diagnostics for the model revealed a pattern characterized by two vertical clusters of points on either side of zero. The loess smooth curve exhibited a slight dip in the central region, which was a geometric artifact of smoothing across these two distinct subgroups. This finding did not indicate model misspecification but rather confirmed that the model adequately captured the major source of heterogeneity in the data. This pattern was attributable to the strong, dichotomous effect of gender on hemoglobin concentration because when we verified it separately in males (**Panel B**) and females (**Panel C**), this pattern also no longer existed.

In the **Panel B**, the residual plot in the male-specific model revealed four vertical clusters of points, corresponding to the four discrete altitude levels in our sampling design.

The parallel-to-zero pattern of the smooth curves in males and females also demonstrated no systematic bias in our model’s predictions.

Table S1. AIC and BIC for restricted cubic splines models

Mutivariable RCS models	AIC	BIC
**[Hb]-Age (model 1)**		
Three-knot model	**8617.219**	**8657.214**
Four-kont model	8618.537	8663.531
Five-knot model	8620.574	8670.568
**[Hb]-Altitude (model 2)**		
Three-knot model	**8616.128**	**8656.123**
Four-kont model	8618.091	8663.085
Five-knot model	8617.968	8662.962

AIC, Akaike Information Criterion; BIC, Bayesian Information Criterion.

Table S2. Mutivariable restricted cubic splines models

Model	Low	High	Difference	Effect	S.E.	*P*
**Model 1 (three knots)***						
Age (year)	40	58	18	0.097	0.514	0.974
Nonlinear						0.860
Altitude (meter)	3500	4014	514	1.036	0.421	**0.014**
Gender	Male	Female	*NA*	-21.370	0.952	**< 0.001**
Hypertension	No	Yes	*NA*	0.401	0.990	0.685
Pulmonary infection	No	Yes	*NA*	-2.381	0.990	**0.016**
**Model 1 (four knots)**						
Age (year)	40	58	18	0.857	1.057	0.866
Nonlinear						0.702
Altitude (meter)	3500	4014	514	1.030	0.421	**0.015**
Gender	Male	Female	*NA*	-21.442	0.957	**< 0.001**
Hypertension	No	Yes	*NA*	0.377	0.991	0.703
Pulmonary infection	No	Yes	*NA*	-2.367	0.991	**0.017**
**Model 1 (five knots)**						
Age (year)	40	58	18	0.817	1.060	0.952
Nonlinear						0.880
Altitude (meter)	3500	4014	514	1.032	0.421	**0.014**
Gender	Male	Female	*NA*	-21.433	0.958	**< 0.001**
Hypertension	No	Yes	*NA*	0.381	0.991	0.701
Pulmonary infection	No	Yes	*NA*	-2.371	0.991	**0.017**
**Model 2 (three knots)***						
Altitude (meter)	3500	4014	514	1.517	0.618	**0.027**
Nonlinear						0.291
Age (year)	40	58	18	0.095	0.499	0.848
Gender	Male	Female	*NA*	-21.405	0.935	**< 0.001**
Hypertension	No	Yes	*NA*	0.414	0.990	0.676
Pulmonary infection	No	Yes	*NA*	-2.393	0.989	**0.016**
**Model 2 (four knots)**						
Altitude (meter)	3500	4014	514	1.391	0.900	**0.044**
Nonlinear						0.562
Age (year)	40	58	18	0.087	0.500	0.862
Gender	Male	Female	*NA*	-21.400	0.936	**< 0.001**
Hypertension	No	Yes	*NA*	0.423	0.991	0.670
Pulmonary infection	No	Yes	*NA*	-2.391	0.990	**0.016**
**Model 2 (five knots)**						

Altitude (meter)	3500	4014	514	1.201	1.005	**0.041**
Nonlinear						0.529
Age (year)	40	58	18	0.079	0.501	0.874
Gender	Male	Female	*NA*	-21.398	0.936	< **0.001**
Hypertension	No	Yes	*NA*	0.430	0.991	0.664
Pulmonary infection	No	Yes	*NA*	-2.393	0.990	**0.016**

NA, not applicable.

*The three-knot models with the lowest Akaike Information Criterion and Bayesian Information Criterion were selected for final presentations.

Table S3. Comorbidities of **male patients** with HAPC (top 10)

Comorbidities	Number of patients	Prevalence
Hypertension	159	18.2%
Pneumonia*	151	17.2%
Liver disease	89	10.2%
Gastrointestinal disease	82	9.4%
Chronic airway disease†	63	7.2%
Heart failure	63	7.2%
Tuberculosis	53	6.1%
Cerebrovascular disease	48	5.5%
Cholelithiasis	36	4.1%
Pulmonary heart disease	34	3.9%

HAPC, high-altitude polycythemia.

*Pneumonia related to mycobacteria was not included.

†Including chronic obstructive pulmonary disease, chronic bronchitis, bronchiectasis and asthma.

Table S4. Comorbidities of **female patients** with HAPC (top 10)

Comorbidities	Number of patients	Prevalence
Hypertension	44	19.8%
Pneumonia*	42	18.9%
Heart failure	42	18.9%
Chronic airway disease†	35	15.8%
Pulmonary heart disease	29	13.1%
Gastrointestinal disease	25	11.3%
Tuberculosis	23	10.4%
Cerebrovascular disease	14	6.3%
Liver disease	14	6.3%
Cholelithiasis	9	4.1%

HAPC, high-altitude polycythemia.

*Pneumonia related to mycobacteria was not included.

†Including chronic obstructive pulmonary disease, chronic bronchitis, bronchiectasis and asthma.

Table S5. ICD10-CN codes of the top 10 comorbidities

Comorbidities	ICD10-CN codes
Hypertension	I10.x00x002 / I10.x00x022 / I10.x00x027 / I10.x00x031 / I10.x00x032 / I10.x04 (essential / primary hypertension), I15.101 (hypertension secondary to renal disorders)
Pneumonia	J18.900 / J18.901 (acute pneumonitis), J18.805 (post-obstructive pneumonia), J18.201 (hypostatic pneumonia), J15.902 (bacterial pneumonia), J98.402 / J98.414 (pulmonary infection)
Heart failure	I50.900 / I50.900x002 / I50.900x014 (heart failure)
Chronic airway disease	J44.900 / J44.901 (chronic obstructive pulmonary disease), J44.100 / J44.101 (chronic obstructive pulmonary disease with acute exacerbation), J45.900x001 (asthma), J42.x02 (chronic bronchitis), J47.x01 (bronchiectasis)
Pulmonary heart disease	I27.902 (cor pulmonale (chronic))
Gastrointestinal disease	K29.101 (acute gastritis), K29.303 (chronic superficial gastritis), K29.401 / K29.400x001 (chronic atrophic gastritis), K29.500 / K29.502 (chronic gastritis), K29.801 (duodenitis), K25.900x001 / K25.906 (gastric ulcer), K26.901 (duodenal ulcer), K31.814 (gastric perforation), K52.905 / K52.908 (acute noninfective gastroenteritis), K52.914 (chronic noninfective gastroenteritis), K56.702 (ileus)
Tuberculosis	A16.202 (tuberculosis of lung), A16.504 (tuberculous pleurisy), A18.307+ (tuberculous peritonitis), B90.901 / B90.902 (sequelae of tuberculosis)
Cerebrovascular disease	I60.901 (subarachnoid haemorrhage), I61.101 (cerebral lobe haemorrhage), I63.900 / I63.905 (cerebral infarction), I64.x03 (cerebrovascular accident)
Liver disease	R94.501 (abnormal results of liver function studies), K70.300 (alcoholic cirrhosis of liver), K74.100 (cirrhosis of liver), K76.000 (fatty (change of) liver), K76.001 (nonalcoholic fatty liver disease), B16.905 (acute hepatitis B), B18.001 (chronic viral hepatitis B with delta-agent)
Cholelithiasis	K80.000 (calculus of gallbladder with acute cholecystitis), K80.203 (calculus of gallbladder without cholecystitis), K80.501

	(calculus of common bile duct without cholangitis or cholecystitis), K80.502 (calculus of bile duct without cholangitis or cholecystitis)

ICD10-CN: Simplified Chinese version of International Classification of Diseases-10.
